# Supplementary material for: Exploring Safety Culture in the ICU of a Large Acute Teaching Hospital through Triangulating Different Data Sources
Source: Healthcare (Basel). 2023 Dec 4;11(23):3095. doi: 10.3390/healthcare11233095 (PMC10705896; doi:10.3390/healthcare11233095)
Supplement: Supplementary file 1 [file healthcare-11-03095-s001.zip › Supplementary SC EL.pdf]

Supplementary Materials

Supplementary File S1

ICU Adverse Event Trigger Tool Worksheet

(Information pertains only to the patient's stay in the ICU)

Hospital MemberID#	Patient's Reference #	Patient's Age	Patient's Gender
☐☐☐☐☐☐☐☐☐☐☐☐☐	☐☐☐☐	☐☐☐ **yrs**	☐ **F** ☐**M**

Triggers:

Which triggers appeared in the patient's record? (Mark all that apply)

Adverse Events:

***If an adverse event occurred, mark the highest harm category:**

Triggers	No Harm	E	F	G	H	I
☐ Positive blood culture………………………………….	ϒ	ϒ	ϒ	ϒ	ϒ	ϒ
☐ Abrupt drop in Hg > 4gms……………………………..	ϒ	ϒ	ϒ	ϒ	ϒ	ϒ
☐ *C. difficile* positive ……..………………………………	ϒ	ϒ	ϒ	ϒ	ϒ	ϒ
☐ PTT > 100 ………………………………………………	ϒ	ϒ	ϒ	ϒ	ϒ	ϒ
☐ INR > 6 …………………………………………………	ϒ	ϒ	ϒ	ϒ	ϒ	ϒ
☐ Glucose < 50 ……………………………………………	ϒ	ϒ	ϒ	ϒ	ϒ	ϒ
☐ Rising BUN and/or Serum Creatinine to 2x baseline…...	ϒ	ϒ	ϒ	ϒ	ϒ	ϒ
☐ Radiologic tests for emboli or clot……………………..	ϒ	ϒ	ϒ	ϒ	ϒ	ϒ
☐ Benadryl ………………………………………………..	ϒ	ϒ	ϒ	ϒ	ϒ	ϒ
☐ Vitamin K …………………………………………….	ϒ	ϒ	ϒ	ϒ	ϒ	ϒ
☐ Flumazenil (Romazicon) ………………………………	ϒ	ϒ	ϒ	ϒ	ϒ	ϒ
☐ Naloxone (Narcan) ……………………………………..	ϒ	ϒ	ϒ	ϒ	ϒ	ϒ
☐ Antidiarrheals…………………………………………...	ϒ	ϒ	ϒ	ϒ	ϒ	ϒ
☐ Antiemetics……………………………………………..	ϒ	ϒ	ϒ	ϒ	ϒ	ϒ
☐ Sodium Polystyrene (Kayexalate) ………………………	ϒ	ϒ	ϒ	ϒ	ϒ	ϒ
☐ Code …………………………………………………….	ϒ	ϒ	ϒ	ϒ	ϒ	ϒ
☐ Pneumonia onset in unit……………………….………..	ϒ	ϒ	ϒ	ϒ	ϒ	ϒ
☐ Readmission to ICU…………………………………….	ϒ	ϒ	ϒ	ϒ	ϒ	ϒ
☐ New onset dialysis……………………………………..	ϒ	ϒ	ϒ	ϒ	ϒ	ϒ
☐ In-unit procedures………………………………………	ϒ	ϒ	ϒ	ϒ	ϒ	ϒ
☐ Intubation/Reintubation…………………………………	ϒ	ϒ	ϒ	ϒ	ϒ	ϒ
☐ Abrupt medication stop ………………………………...	ϒ	ϒ	ϒ	ϒ	ϒ	ϒ
☐ Oversedation/Lethargy/Hypotension…………………..	ϒ	ϒ	ϒ	ϒ	ϒ	ϒ
☐ Other…………………………………….……………...	ϒ	ϒ	ϒ	ϒ	ϒ	ϒ
	ϒ	ϒ	ϒ	ϒ	ϒ	ϒ

©VHA/Institute for Healthcare Improvement, January 2002

ICU Length of Stay ϒϒϒ days

Total Number of <u>Distinct</u> Adverse Events ϒ

Fill out the following information below, for <u>each DISTINCT</u> adverse event with a <u>harm category of E through I</u>:

	**Harm Category* (E/F/G/H/I)**	**Medication Related (Y/N)**	**Type of Medication** (0 to 7)**	**"E" coded in UB92*** (Y/N)**
Event **#1**……	ϒ	ϒ	ϒ	ϒ
Event **#2**……	ϒ	ϒ	ϒ	ϒ
Event **#3**……	ϒ	ϒ	ϒ	ϒ
Event **#4**……	ϒ	ϒ	ϒ	ϒ
Event **#5**……	ϒ	ϒ	ϒ	ϒ
Event **#6**……	ϒ	ϒ	ϒ	ϒ
Event **#7**……	ϒ	ϒ	ϒ	ϒ

<u>Harm Categories</u>
Category E: Contributed to temporary harm to the patient and required intervention
Category F: Contributed to temporary harm to the patient and required initial/prolonged hospitalization
Category G: Ccontributed to permanent patient harm
Category H: Required intervention to sustain life
Category I: Contributed to the patient's death

****<u>Medication Code</u>***
0—Not applicable
1—Antibiotics
2—Anticoagulants and Antiplatelets
3—Antipsychotics
4—Electrolytes
5—Insulin 6—
Narcotics 7—
Sedatives

"<u>E" Code in UB92 Form</u>
*Was the adverse event documented as an **"E" code** in the ICD9 section of the UB92 form?*
Yes—only pertains to documentation of the following "E" codes: Accidental poisoning (E850-E869); Therapeutic use (E930-E949); Suicide attempt (E950-952); Assault(E961-E962); Undetermined (E980-E982)

Hospital Survey on Patient Safety (Version 2.0)

Instructions

This survey asks for your opinions about patient safety issues, medical error, and event reporting inyour hospital and will take about 10-15 minutes to complete. If a question does not apply to you or your hospital or you don't know the answer, please select "Does Not Apply or Don't Know."

- *"**Patient safety**" is defined as the avoidance and prevention of patient injuriesor adverse events resulting from the processes of healthcare delivery.*
- *A "**patient safety event**" is defined as any type of healthcare-related error, mistake, or incident, regardless of whether or not it results in patient harm.*

Your Staff Position

1. **What is your position in this hospital?**

Select ONE answer.

Nursing

☐ 1 Advanced Practice Nurse (ANP, CNS, CNM,CNF)
☐3 Health Care Assistant
☐4 Registered Nurse (RN)

Medical

☐6 Intern/ Senior House Officer
☐7 Registrar / Consultant

Other Clinical Position

☐10 Health and Social Care Professional

Supervisor, Manager, Clinical Leader, Senior Leader

☐ 15 Supervisor, Manager, Department Manager,Clinical Leader, Administrator, Director
☐16 Senior Leader, Executive, C-Suite

Support

☐17 Facilities
☐18 Food Services
☐19 Housekeeping, Environmental Services
☐20 Information Technology, Health InformationServices, Clinical Informatics
☐21 Security
☐22 Transporter
☐23 Unit Clerk, Secretary, Receptionist, Office Staff

Other

☐24 Other, please specify:

Your Unit/Work Area

2. **Think of your "unit" as the work area, department, or clinical area of the hospital where you spend** *most* of your work time. What is your primary unit or work area in this hospital?

Select ONE answer.

Multiple Units, No specific unit
- ☐1 Many different hospital units,No specific unit

Medical/Surgical Units
- ☐2 Combined Medical/SurgicalUnit
- ☐3 Medical Unit (Non-Surgical)
- ☐4 Surgical Unit

Patient Care Units
- ☐5 Cardiology
- ☐6 Emergency Department, Observation, Short Stay
- ☐7 Gastroenterology
- ☐8 ICU (all adult types)
- ☐9 Labor & Delivery, Obstetrics & Gynecology
- ☐10 Oncology, Hematology
- ☐11 Pediatrics (including NICU,PICU)
- ☐12 Psychiatry, Behavioral Health
- ☐13 Pulmonology
- ☐14 Rehabilitation, Physical Medicine
- ☐15 Telemetry

Surgical Services
- ☐16 Anesthesiology
- ☐17 Endoscopy, Colonoscopy
- ☐ 18 Pre Op, Operating Room/Suite, PACU/Post Op, Peri Op

Clinical Services
- ☐19 Pathology, Lab
- ☐20 Pharmacy
- ☐21 Radiology, Imaging
- ☐22 Respiratory Therapy
- ☐ 23 Social Services, Case Management, Discharge Planning

Administration/Management
- ☐24 Administration, Management
- ☐25 Financial Services, Billing
- ☐26 Human Resources, Training
- ☐ 27 Information Technology,Health Information Management, Clinical Informatics
- ☐ 28 Quality, Risk Management,Patient Safety

Support Services
- ☐29 Admitting/Registration
- ☐30 Food Services, Dietary
- ☐ 31 Housekeeping, Environmental Services, Facilities
- ☐32 Security Services
- ☐33 Transport

Other
- ☐34 Other, please specify:

SECTION A: Your Unit/Work Area

How much do you agree or disagree with the following statements about your unit/work area?

**Think about your unit/work area:**	**Strongly Disagree** ▼	**Disagree** ▼	**Neither Agree nor Disagree** ▼	**Agree** ▼	**Strongly Agree** ▼	**Does Not Apply or Don't Know** ▼
1. In this unit, we work together as an effective team..	☐1	☐2	☐3	☐4	☐5	☐9
2. In this unit, we have enough staff to handle the workload	☐1	☐2	☐3	☐4	☐5	☐9
3. Staff in this unit work longer hours than is best for patient care	☐1	☐2	☐3	☐4	☐5	☐9
4. This unit regularly reviews work processes to determine if changes are needed to improve patient safety	☐1	☐2	☐3	☐4	☐5	☐9
5. This unit relies too much on temporary, float, or PRN staff	☐1	☐2	☐3	☐4	☐5	☐9
6. In this unit, staff feel like their mistakes are held against them	☐1	☐2	☐3	☐4	☐5	☐9
7. When an event is reported in this unit, it feels like the person is being written up, not the problem...	☐1	☐2	☐3	☐4	☐5	☐9
8. During busy times, staff in this unit help each other	☐1	☐2	☐3	☐4	☐5	☐9
9. There is a problem with disrespectful behavior by those working in this unit	☐1	☐2	☐3	☐4	☐5	☐9
10. When staff make errors, this unit focuses on learning rather than blaming individuals...........	☐1	☐2	☐3	☐4	☐5	☐9
11. The work pace in this unit is so rushed that it negatively affects patient safety	☐1	☐2	☐3	☐4	☐5	☐9
12. In this unit, changes to improve patient safety are evaluated to see how well they worked	☐1	☐2	☐3	☐4	☐5	☐9
13. In this unit, there is a lack of support for staff involved in patient safety errors	☐1	☐2	☐3	☐4	☐5	☐9
14. This unit lets the same patient safety problems keep happening	☐1	☐2	☐3	☐4	☐5	☐9

SECTION B: Your Supervisor, Manager, or Clinical Leader

How much do you agree or disagree with the following statements about your immediate supervisor,manager, or clinical leader?

	Strongly Disagree ▼	Disagree ▼	Neither Agree nor Disagree ▼	Agree ▼	Strongly Agree ▼	Does Not Apply or Don't Know ▼
1. My supervisor, manager, or clinical leader seriously considers staff suggestions for improving patient safety	☐1	☐2	☐3	☐4	☐5	☐9
2. My supervisor, manager, or clinical leader wants us to work faster during busy times, even if it means taking shortcuts	☐1	☐2	☐3	☐4	☐5	☐9
3. My supervisor, manager, or clinical leader takes action to address patient safety concerns that are brought to their attention	☐1	☐2	☐3	☐4	☐5	☐9

SECTION C: Communication

How often do the following things happen in your unit/work area?

**Think about your unit/work area:**	Never ▼	Rarely ▼	Some-times ▼	Most of the time ▼	Always ▼	Does Not Apply or Don't Know ▼
1. We are informed about errors that happen in this unit	☐1	☐2	☐3	☐4	☐5	☐9
2. When errors happen in this unit, we discuss ways to prevent them from happening again ..	☐1	☐2	☐3	☐4	☐5	☐9
3. In this unit, we are informed about changes that are made based on event reports	☐1	☐2	☐3	☐4	☐5	☐9
4. In this unit, staff speak up if they see something that may negatively affect patient care	☐1	☐2	☐3	☐4	☐5	☐9
5. When staff in this unit see someone with more authority doing something unsafe for patients, they speak up	☐1	☐2	☐3	☐4	☐5	☐9
6. When staff in this unit speak up, those with more authority are open to their patient safety concerns	☐1	☐2	☐3	☐4	☐5	☐9
7. In this unit, staff are afraid to ask questions when something does not seem right	☐1	☐2	☐3	☐4	☐5	☐9

SECTION D: Reporting Patient Safety Events

Think about your unit/work area:	Never ▼	Rarely ▼	Some-times ▼	Most of the time ▼	Always ▼	Does Not Apply or Don't Know ▼
1. When a mistake is caught and corrected before reaching the patient, how often is this reported?	☐1	☐2	☐3	☐4	☐5	☐9
2. When a mistake reaches the patient and could have harmed the patient, but did not, how often is this reported?	☐1	☐2	☐3	☐4	☐5	☐9

3. In the past 12 months, how many patient safety events have you reported?

☐a. None
☐b. 1 to 2
☐c. 3 to 5
☐d. 6 to 10
☐e. 11 or more

SECTION E: Patient Safety Rating

1. How would you rate your unit/work area on patient safety?

Poor ▼	Fair ▼	Good ▼	Very Good ▼	Excellent ▼
☐1	☐2	☐3	☐4	☐5

SECTION F: Your Hospital

How much do you agree or disagree with the following statements about your hospital?

Think about your hospital:	Strongly Disagree ▼	Disagree ▼	Neither Agree nor Disagree ▼	Agree ▼	Strongly Agree ▼	Does Not Apply or Don't Know ▼
1. The actions of hospital management show that patient safety is a top priority	☐1	☐2	☐3	☐4	☐5	☐9
2. Hospital management provides adequate resources to improve patient safety	☐1	☐2	☐3	☐4	☐5	☐9
3. Hospital management seems interested in patient safety only after an adverse event happens..	☐1	☐2	☐3	☐4	☐5	☐9
4. When transferring patients from one unit to another, important information is often left out.	☐1	☐2	☐3	☐4	☐5	☐9

5. During shift changes, important patient care information is often left out	☐1	☐2	☐3	☐4	☐5	☐9
6. During shift changes, there is adequate time to exchange all key patient care information ...	☐1	☐2	☐3	☐4	☐5	☐9

Background Questions

1. How long have you worked in this <u>hospital</u>?

☐a. Less than 1 year
☐b. 1 to 5 years
☐c. 6 to 10 years
☐d. 11 or more years

2. In this hospital, how long have you worked in your current <u>unit/work area</u>?

☐a. Less than 1 year
☐b. 1 to 5 years
☐c. 6 to 10 years
☐d. 11 or more years

3. Typically, how many hours per week do you work in this <u>hospital</u>?

☐a. Less than 30 hours per week
☐b. 30 to 40 hours per week
☐c. More than 40 hours per week

4. In your staff position, do you typically have direct interaction or contact with patients?

☐a. YES, I typically have direct interaction or contact with patients
☐b. NO, I typically do NOT have direct interaction or contact with patients

Your Comments

Please feel free to provide any comments about how things are done or could be done in yourhospital that might affect patient safety.

Thank you for completing this survey
